# Data on growth performance and nutritional composition of common carp (*Cyprinus carpio*) fry fed with *Chlamydomonas* sp.

**DOI:** 10.1016/j.dib.2024.110494

**Published:** 2024-05-08

**Authors:** Sumit Kanti Dey, Jannatul Nayeem, Proma Dey, Abed Hasan Tuser, Inkiad Ahmed Himel, Razia Sultana, Mohammed Nurul Absar Khan, Helena Khatoon

**Affiliations:** Faculty of Fisheries, Chattogram Veterinary and Animal Sciences University, Chattogram 4225, Bangladesh

**Keywords:** Common carp, *Chlamydomonas* sp., Growth performance, Proximate composition, Fatty acid, Amino acid

## Abstract

A feeding trial spanning two months was conducted to evaluate the effects of *Chlamydomonas* sp*.* on growth performance, water quality, survival, proximate composition and biochemical profile of common carp (*Cyprinus carpio*) where fishmeal was partially replaced using *Chlamydomonas* sp*.* in the diet. Twenty uniform-sized common carp fries were distributed into triplicate groups and placed in 40-liter rectangular glass tanks. *Chlamydomonas* sp. was added at different levels in the diet: 0 % (control), 5 % (C5), 10 % (C10), 15 % (C15) along with the commercial feed (CMF). After the experiment, random sampling of fish was performed to conduct all the analyses. Significant variations (*p* < 0.05) were found in the chemical water quality parameters. The highest survival rate was recorded from C15 (81.67 %) followed by C10 (71.67 %), CMF (63.33 %) and C5 (58.33 %) respectively compared with the control (43.2 %). There were significant differences (*p* < 0.05) observed among all the treatments in terms of Average Daily Gains (ADG), Specific Growth Rate (SGR) and length increments. Protein content ranged the highest and the lowest in C15 (35.6 %) and control (24.8 %) respectively. The C15 group showed the highest lipid content (15.7 %) and the control group showed the lowest lipid (8.4 %). The inclusion of *Chlamydomonas* sp*.* in the diet had a significant positive impact on the fatty acid and amino acid profile of whole common carp. Present data revealed that substituting a portion of fishmeal with *Chlamydomonas* sp. powder could provide higher growth performance, offering both nutritional benefits and higher survival rate in common carp (*Cyprinus carpio*).

Specifications Table

**Table:** 

Subject	Food Science, Aquatic Science
Specific subject area	Effects of microalgae formulated feed on fish growth, survival, proximate composition, water quality, fatty acid and amino acid composition
Data format	Raw and analyzed primary data
Type of data	Graph and Table
Data collection	For growth performance of common carp fry: Length increment, average daily gain and specific growth rate was calculated using the formula outlined by Ayala et al. (2020). For survival rate: Mortality Data of physical parameters of culture water such as- dissolved oxygen, temperature, pH were collected with glass thermometer, a portable pH meter (pHep-HI98107, HANNA, Romania) and dissolved oxygen meter (DO-5509, Lutron). Chemical parameters (NO_2__—_N, TAN and SRP) were determined following the methods of Parsons et al. (1984) by using a spectrophotometer. Fatty acid and amino acid composition were determined according to the methods of Griffiths et al. (2010), GCMS (SHIMADZU, Japan) and the Moore and Stein method (1951) respectively. The collected data were analyzed by using MS Excel and IBM SPSS (v. 26.0) software.
Data source location	Disease and Microbiology Laboratory, Department of Aquaculture, Chattogram Veterinary and Animal Sciences University (CVASU), Khulshi-4225, Chattogram, Bangladesh
Data accessibility	Data are available with this article and also at Repository name: Mendeley Data Data identification number: Direct URL to data: https://data.mendeley.com/datasets/7x9z7wr5pc/1

## 1 Value of the Data

• The data on growth performance, survival rate and proximate composition of common carp fry will encourage the feed formulators to prioritize microalgal inclusion in fish diet to achieve greater growth and nutritional performance.
• Inclusion of microalgae can also be utilized to improve the water quality of the fish tanks. Microalgae not only elevate the levels of protein, lipids, essential fatty acids, and amino acids in fish but also contribute to improved growth and gonadal development, thereby positively impacting consumer health.
• These data contribute to enhancing fish quality and production through higher survival, productivity and nutritional properties of commercial fish in the aquaculture sector.

## 2 Background

The demand for fish meal in aquafeed production has witnessed a 300 % increase over the last ten years as it serves as a key feed ingredient [2]. Limited and unpredictable supply of fish meal and fish oil from wild fish led industries and researchers to explore sustainable alternatives for fishmeal [2]. Microalgae present a more sustainable source of aquafeed when compared to fishmeal and fish oil [3], contributing positively to the growth, disease resistance, skin coloration, and physiological activity of the fish [4]. *Chlamydomonas* sp. exhibits a superior nutritional profile compared to commercial microalgal species like *Chlorella* and *Spirulina* [5]. Due to its exceptional nutritional qualities, this species has substantial potential as either a new superfood or as a valuable component in dietary supplements. However, comprehensive research about the effects of *Chlamydomonas* sp. on the diets of common carp has not been conducted yet. Therefore, this study aimed to evaluate the efficiency of *Chlamydomonas* sp. supplementation at different levels on the growth performance, proximate composition and biochemical profile of common carp.

## 3 Data Description

The growth performance of common carp (*Cyprinus carpio*) fry fed with *Chlamydomonas* sp. is presented in this dataset along with water quality parameters, proximate and biochemical composition [1].

The findings of physical parameters including temperature, DO and pH in the different treatments showed no significant differences (*p* < 0.05) (Table 1). Chemical parameters such as Nitrite Nitrogen (NO_2__—_N), Total Ammonia Nitrogen (TAN) and Soluble Reactive Phosphorus (SRP) of different treatments had significant differences (*p* < 0.05) (Table 2). The highest NO_2__—_N, TAN and SRP concentrations were obtained from control group about 0.15±0.01 mg/L, 0.65±0.01 mg/L and 0.49±0.01 mg/L respectively. Lowest NO_2__—_N, TAN and SRP were obtained from C10 about 0.05±0.01, 0.42±0.01 and 0.33±0.01.

**Table 1:** Physical parameters recorded from common carp fry rearing tanks during the experimental period (mean ±SE).

Treatment	Parameter		
	Temperature (°C)	DO (mg L^−1^)	pH
Control	27.92 ± 0.33^a^	6.43 ± 0.27^a^	8.32 ± 0.09^a^
5 %	27.64 ± 0.28^a^	6.60 ± 0.1^a^	8.27 ± 0.07^a^
10 %	27.73 ± 0.16^a^	6.53 ± 0.15^a^	8.18 ± 0.14^a^
15 %	27.97 ± 0.54^a^	6.57 ± 0.09^a^	7.92 ± 0.10^a^
CMF	27.44 ± 0.17^a^	6.17 ± 0.27^a^	8.24 ± 0.08^a^

**Table 2:** Chemical parameters obtained from common carp fry rearing tanks during the experimental period. Values are mean ± Standard error.

Treatment	Chemical parameter		
	NO_2_-N (mg/L)	TAN (mg/L)	SRP (mg/L)
Control	0.15 ± 0.01^a^	0.65 ± 0.01^a^	0.49 ± 0.01^a^
C5	0.07 ± 0.01^d^	0.49 ± 0.01^d^	0.35 ± 0.01^d^
C10	0.05 ± 0.00^e^	0.42 ± 0.01^e^	0.33 ± 0.01^e^
15 %	0.09 ± 0.00^b^	0.58 ± 0.01^b^	0.40 ± 0.01^b^
CMF	0.08 ± 0.01^c^	0.53 ± 0.01^c^	0.37 ± 0.01^c^

**Fig. 1:**
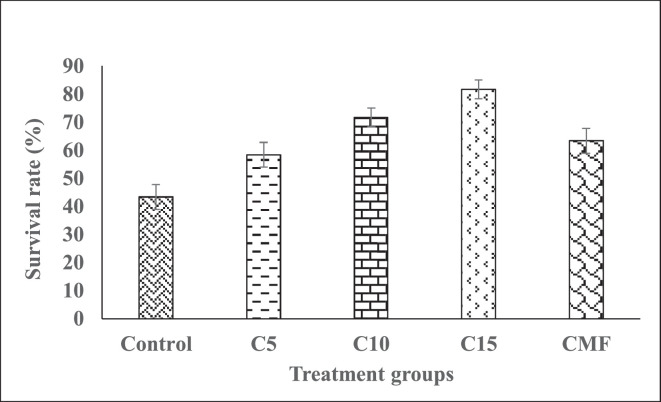
Survival rate (%) of common carp (*Cyprinus carpio*) fry fed with different concentrations of *Chlamydomonas* sp. (means ± SE).

Significant variations in the survival rates of different treatments are depicted in Fig. 1. Survival rate was found higher in the treatment groups fed with *Chlamydomonas* sp*.* incorporated diets including C15 followed by C10, CMF and C5 respectively compared with the control group (Table 3).

**Table 3:** Different growth indices of common carp fry fed with experimental diets. Values are means ± SE of three replicate groups (*n* = 3). Here, LI, Length increment; ADG, Average daily gain; SGR, Specific growth rate.

Diet					
	Control	C5	C10	C15	CMF
Initial length (cm)	1.83 ± 0.03^a^	1.83 ± 0.02^a^	1.82 ± 0.01^a^	1.83 ± 0.01^a^	1.82 ± 0.01^a^
Final length (cm)	2.79 ± 0.04^e^	2.92 ± 0.03^d^	3.32 ± 06^b^	3.49 ± 06^a^	3.24 ± 0.03^c^
LI (cm)	0.97 ± 0.07^e^	1.09 ± 0.02^d^	1.50 ± 0.03^b^	1.66 ± 0.06^a^	1.41± 0.02^c^
Initial weight (g)	0.24 ± 0.01^a^	0.29± 0.01^a^	0.28 ± 0.00^a^	0.23 ± 0.01^a^	0.31 ± 0.01^a^
Final weight (g)	0.78 ± 0.03^e^	0.95± 0.01^d^	1.19 ± 0.02^b^	1.25 ± 0.04^a^	1.03 ± 0.03^c^
ADG (g)	0.01 ± 0.00^e^	0.01 ± 0.00^d^	0.02 ± 0.00^b^	0.02 ± 0.00^a^	0.01 ± 0.00^c^
SGR (%)	1.94 ± 0.06^e^	2.00 ± 0.01^d^	2.41± 0.04^b^	2.85 ± 0.13^a^	2.03 ± 0.02^c^

**Fig. 2:**
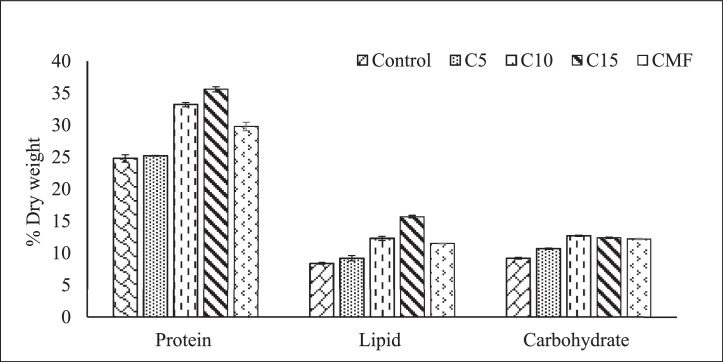
Proximate composition of whole fish body (*Cyprinus carpio*) fed with different concentrations of *Chlamydomonas* sp. Here values are means of triplicates ± SE.

All the treatment groups showed significant differences in protein, lipid and carbohydrate content (Fig. 2). The protein content ranges from C15(35.6 %) and control (24.8 %) group. C15 group had the highest lipid content (15.7 %) and control group had the lowest value (8.4 %). Results also indicate that carbohydrate level was significantly lower in control (9.2 %) and higher in C10 (12.7 %) treatment group.

The inclusion of *Chlamydomonas* sp*.* in the diet had a significant positive impact on the fatty acid composition of common carp (*Cyprinus carpio*). The highest PUFA along with higher n-3 and n-6 fatty acids were predominant in the C15 treatment. Essential PUFAs like linoleic acid (18:2n-6), EPA (C20:5n-3) and DHA (C22:6n-3) were found dominant in C15, C10 and CMF treatment respectively (Table 4).

**Table 4:** Fatty acid composition of Common Carp (*Cyprinus carpio*) fry fed with different experimental diets (Control, C5, C10, C15, CMF). Values are expressed as mean ±SE.

Carbon	Fatty Acid Methyl Esters	Control	C5	C10	C15	CMF
		Conc. (ppm)				
C8:0	Methyl Octanoate	1.26±0.02	0.05±0.00	0.06±0.00	0.73±0.01	1.47±0.00
C10:0	Methyl Decanoate	1.02±0.01	4.48±0.20	1.18±0.05	0.48±0.00	0.50±0.09
C12:0	Methyl Laurate	4.88±0.05	0.04±0.00	5.89±0.08	1.99±0.03	6.37±0.11
C13:0	Methyl Tridecanoate	2.49±0.00	2.30±0.09	0.53±0.01	0.28±0.00	0.42±0.01
C14:0	Methyl Myristate	2.14±0.00	3.47±0.15	0.17±0.00	0.08±0.01	1.31±0.04
C16:0	Methyl Palmitate	18.66±0.09	15.33±0.67	9.28±0.09	8.56±0.10	15.76±0.09
C18:0	Methyl Stearate	0.09±0.01	0.14±0.03	3.13±0.15	2.67±0.03	0.23±0.01
C20:0	Methyl Arachidate	5.20±0.15	5.84±0.02	4.85±0.19	7.98±0.76	4.17±0.11
C17:0	Methyl Heptadecanoate	0.02±0.01	0.04±0.00	0.08±0.07	2.79±0.02	0.08±0.01
C21:0	Methyl Heneicosanoate	2.16±0.02	3.57±0.09	4.43±0.18	0.04±0.00	1.51±0.20
C22:0	Methyl Behenate	ND±ND	0.51±0.04	1.13±0.03	2.75±0.05	1.66±0.06
C23:0	Methyl Tricosanoate	ND±ND	ND±ND	ND±ND	0.06±0.01	ND±ND
C24:0	Methyl Lignocerate	ND±ND	ND±ND	ND±ND	ND±ND	ND±ND
	**ƩSAFA**	**37.92±0.02^a^**	**35.77±0.82^ab^**	**30.74±0.56^cd^**	**28.41±0.76^d^**	**33.48±0.18^bc^**
C16:1	Methyl Palmitoleate	26.02±0.22	25.17±0.09	35.48±0.97	39.72±0.50	29.79±0.09
C18:1	Methyl Oleate	6.38±0.00	7.10±0.06	4.83±0.01	0.47±0.02	4.50±0.06
C20:1	Methyl cis-11-eicosenoate	0.01±0.01	0.04±0.02	0.08±0.01	0.15±0.02	0.01±0.00
C22:1	Methyl Erucate	3.10±0.03	4.47±0.18	1.12±0.02	0.26±0.00	3.29±0.11
C24:1	Methyl Nervonate	0.02±0.01	0.21±0.11	0.05±0.03	0.01±0.00	0.02±0.01
	**ƩMUFA**	**35.53±0.23^b^**	**36.98±0.33^b^**	**41.56±0.93^a^**	**40.62±0.49^a^**	**37.60±0.08^b^**
C18:2n-6	Methyl Linoleate	10.14±0.11	11.39±0.61	12.19±0.91	13.11±0.05	10.29±0.06
C20:3n-6	Methyl 11-14-17- Eicosatrienoate	0.62±0.01	0.15±0.06	0.84±0.04	1.44±0.10	0.89±0.02
C20:4n-6	Methyl Arachidonate	0.13±0.01	0.49±0.03	0.89±0.02	0.43±0.02	0.27±0.00
	**Ʃn6-PUFA**	**10.89±0.12^c^**	**12.03±0.53^bc^**	**13.92±0.97^ab^**	**14.98±0.17^a^**	**11.45±0.08^bc^**
C18:3n-3	Methyl Linolenate	1.29±0.06	0.25±0.00	1.47±0.04	4.67±0.03	2.99±0.03
C20:5n-3	Methyl icosa-5,8,11,14,17-pentaenoate	1.03±0.08	0.03±0.00	1.07±0.01	1.86±0.05	1.46±0.06
C22:5n-3	Methyl Docosapentaenoate	11.01±0.12	13.00±0.20	10.66±0.08	9.01±0.01	12.04±0.01
C22:6n-3	Methyl Docosahexanoate	0.16±0.00	0.14±0.00	0.25±0.10	0.11±0.01	0.12±0.03
	**Ʃn3-PUFA**	**13.49±0.02^b^**	**13.42±0.20^b^**	**13.45±0.23^b^**	**15.65±0.03^a^**	**15.61±0.01^a^**
	**ƩPUFA**	**24.38±0.14^b^**	**25.45±0.73^b^**	**27.37±1.20^ab^**	**30.63±0.14^a^**	**27.06±0.07^ab^**
	**Ʃn3/ Ʃn6**	**1.24±0.01^ab^**	**1.12±0.03^bc^**	**0.97±0.05^c^**	**1.04±0.01^c^**	**1.36±0.01^a^**
	**DHA/EPA**	**0.15±0.01^a^**	**5.23±0.24^b^**	**0.23±0.09^b^**	**0.06±0.01^b^**	**0.08±0.02^b^**
	**SAFA/TUFA**	**0.63±0.00^a^**	**0.57±0.01^b^**	**0.45±0.01^d^**	**0.40±0.01^d^**	**0.52±0.00^c^**
	**SAFA/TFA**	**0.39±0.00^a^**	**0.36±0.00^b^**	**0.31±0.00^d^**	**0.29±0.01^e^**	**0.34±0.00^c^**
	**TUFA/TFA**	**0.61±0.00^e^**	**0.64±0.00^d^**	**0.69±0.00^b^**	**0.71±0.01^a^**	**0.66±0.00^c^**

Here, **SAFA** means Saturated Fatty Acids, **MUFA**= Monounsaturated fatty acids, **n6-PUFA**= ω−6 polyunsaturated fatty acids, **n3-PUFA**= ω−3 polyunsaturated fatty acids, **DHA**= Docosahexaenoic acid, **EPA=** Eicosapentaenoic acid, **TUFA**= Total unsaturated fatty acids, **TFA**= Total fatty acids.

Essential and non-essential amino acid variations in the muscle of common carp after two months of feeding trial are presented in Table 5. The highest and lowest essential amino acids were found in C15 (49.57 %) followed by C10 (48.94 %), CMF (47.54 %), C5 (47.23 %) and control (47.07 %). Among the essential amino acids in *Cyprinus carpio*, Valine was predominant in C15(10.44 %). Highest non-essential amino acid was observed in the control group (52.94 %). Aspartic acid (13.31 %), was prevalently found in the control fish groups.

**Table 5:** Amino acid composition of Common Carp (*Cyprinus carpio*) fry fed with *Chlamydomonas* sp. incorporated experimental diets (Control, C5, C10, C15, CMF). Values are mean ±SE (Standard Error).

Compound name	Types	Common Carp (*Cyprinus carpio*)				
		Control	C5	C10	C15	CMF
		Amount (%)				
Histidine	EAA	6.79±0.03	6.78±0.01	7.88±0.02	7.59±0.06	7.33±0.07
Isoleucine	EAA	4.60±0.02	4.61±0.02	4.41±0.03	3.90±0.02	5.42±0.00
Leucine	EAA	4.63±0.01	4.72±0.01	5.47±0.05	5.31±0.10	4.85±0.05
Lysine	EAA	4.14±0.02	4.11±0.12	2.05±0.00	2.22±0.27	2.01±0.00
Methionine	EAA	3.01±0.01	3.19±0.07	3.80±0.02	3.77±0.14	3.83±0.05
Phenylalanine	EAA	3.05±0.02	2.98±0.02	4.24±0.04	4.48±0.08	3.23±0.09
Threonine	EAA	5.38±0.01	4.85±0.03	4.21±0.10	4.45±0.04	4.86±0.02
Tyrosine	EAA	6.37±0.04	6.56±0.04	6.89±0.10	7.05±0.05	6.37±0.05
Valine	EAA	9.16±0.02	9.43±0.01	9.99±0.09	10.44±0.06	9.56±0.04
Alanine	NEAA	5.57±0.01	5.64±0.00	5.08±0.02	5.47±0.02	5.65±0.02
Arginine	NEAA	8.19±0.00	8.03±0.03	10.27±0.03	9.85±0.05	8.87±0.05
Aspartic acid	NEAA	13.22±0.01	13.18±0.03	11.56±0.03	11.16±0.03	12.55±0.03
Glutamic acid	NEAA	5.91±0.00	5.96±0.04	4.94±0.02	5.18±0.00	5.65±0.00
Glycine	NEAA	6.13±0.01	6.21±0.01	4.62±0.01	4.61±0.05	5.98±0.07
Cysteine	NEAA	0.72±0.02	0.44±0.00	0.09±0.01	0.14±0.01	0.24±0.00
Serine	NEAA	4.86±0.00	5.32±0.01	4.63±0.00	4.43±0.02	4.70±0.01
Proline	NEAA	8.29±0.03	7.99±0.01	9.86±0.03	9.52±0.00	8.90±0.06
**∑EAA** **∑NEAA**		**47.11±0.01^b^**	**47.22±0.07^b^**	**48.94±0.01^a^**	**49.19±0.3^a^**	**47.46±0.09^b^**
		**52.88±0.01^a^**	**52.76±0.04^ab^**	**51.03±0.03^c^**	**50.35±0.07^d^**	**52.53±0.09^b^**

## 4 Experimental Design, Materials and Methods

### 4.1 *Collection and culture of Chlamydomonas* sp

Pure isolate of *Chlamydomonas* sp. was obtained from Live Feed Research Corner, Department of Aquaculture, Chattogram Veterinary and Animal Sciences University, Khulshi, Chattogram, Bangladesh. 100 ml stock of *Chlamydomonas* sp. was inoculated in the 900 ml Conway medium [6] to maintain the stock culture. Then the cultures were incubated in controlled indoor conditions for 14 days at 24 °C temperature. Cool inflorescence lamps with 2000 lux light intensity were installed in the room to provide a 12 h light: 12 h dark regime [7]. The stock culture was scaled up to 5 L and transferred to 20 L plastic tanks for mass culture to reach about 16 L culture. Continuous aeration was provided through air pumps and each tank was examined for contamination on weekly basis.

### 4.2 Harvest of microalgae

After 15 days, the cultures of *Chlamydomonas* sp. were harvested by centrifuging at 5000 rpm for 5 min using a centrifuge machine (TL5R Free Standing low speed refrigerated centrifuge, Herexi) and dried in a hot air oven (JSR Korea's Natural Convention Oven LNO-150) at 40 °C temperature for 12 h. The dried biomass was ground using mortar and pestle and sieved. Finely powdered dried biomass was stored in glass vials at 4 °C for the production of test diets.

### 4.3 Experimental design

#### 4.3.1 Test diet preparation

All the formulated test diets were almost equal in protein and lipid content, maintaining a consistent level of crude protein at 30 % and lipid at 6 %. The required feed ingredients along with commercial feed were purchased from local stores in Chattogram, Bangladesh. Feed ingredients were crushed in the grinder and sieved to make fine powder. Then proximate composition of the feed ingredients along with *Chlamydomonas* sp. powder was performed to calculate their percentages for preparing formulated feed. The crude protein, lipid and carbohydrate content of *Chlamydomonas* sp. were found to be 59.51±0.78 %, 21.07±0.71 % and 7.49±0.46 %. According to Table 6, feed ingredients were mixed and then *Chlamydomonas* sp. powder was incorporated into the mixture as needed. To facilitate comparative analysis, dried *Chlamydomonas* sp. powder was integrated into the diet in the following manner: Control (no inclusion), C5 (5 %), C10 (10 %), and C15 (15 %). After mixing all the ingredients, adequate water was added to make a doughy texture. Then the dough was processed to make uniform granular-size feed. Crude protein (Kjeldahl Auto System, ISO 5983–1987) and crude lipid (Soxtec HT6) of the experimental diets along with the commercial diet were also performed to ensure nutritional homogeneity. Feeds were packed into sealed plastic bags, placed in labeled airtight containers and stored in a cool, dry place at 4 °C, away from direct sunlight to maintain feed quality and prevent mold formation.

**Table 6:** Ingredient percentage and proximate composition of the experimental diets.

Dietary group					
Control		Microalgae diet			Commercial
	C	C5	C10	C15	CMF
**Ingredients**					
Fish Meal	25 %	22 %	19 %	16 %	–
Soybean	30 %	28 %	25 %	22 %	–
Wheat Bran	13 %	12 %	11 %	10 %	–
Wheat Flour	8 %	9 %	10 %	10 %	–
Barley Meal	10 %	10 %	10 %	10 %	–
Corn Meal	9 %	9 %	9 %	10 %	–
Starch	3 %	3 %	4 %	5 %	–
Mineral Premix	2 %	2 %	2 %	2 %	–
*Chlamydomonas* sp.	–	5 %	10 %	15 %	–
**Proximate composition**					
Crude protein (%)	30.68 %	30.74 %	30.75 %	30.83 %	30.81 %
Lipid (%)	6.39 %	6.47 %	6.45 %	6.53 %	6.42 %

#### 4.3.2 Collection and stocking of fish

Common carp fries (*Cyprinus carpio*) were purchased from a hatchery located in Mymensingh district in Bangladesh and brought to the lab in aerated plastic bags. Fries were gently released in the tanks and then properly conditioned for 3 days. To disinfect the fries, Potassium permanganate (KMnO_4_) was used. The fries were partitioned into five distinct treatment groups, and within each group, there existed a triplicate replication cohort. Subsequently, every group was placed in a rectangular glass tank (18 × 12 × 14 inches), filled with 40 liters of water and accommodated 20 fries within each replication.

#### 4.3.3 Feeding and monitoring

The fish were provided with test diets at a rate of 5 % of their body weight, administered twice a day in equally divided portions with a six-hour gap between each feeding. To maintain water quality, one-third of the water was siphoned from each tank bottom and fresh water was added to compensate for the removed water. Complete water exchange was also performed in the tanks twice per week. Mortality rate was monitored daily. and Routine monitoring was conducted to detect any instances of stress or disease outbreaks. This study was carried out for consecutive 60 days.

### 4.4 Data collection and analysis

#### 4.4.1 Water quality analysis

Physical parameters of the culture including temperature, DO and pH were measured daily with a glass thermometer, dissolved oxygen meter (DO-5509, Lutron) and portable pH meter (pHep-HI98107, HANNA, Romania) respectively. Chemical parameters were measured weekly. Nitrite-nitrogen (NO_2__—_N), Total ammonia nitrogen (TAN) and Soluble Reactive Phosphorous (SRP) were measured according to Parsons et al. [8].

#### 4.4.2 Analysis of growth performance

The assessment of fish growth performance involved the use of metrics such as average daily gain, specific growth rate, length increment. These growth parameters were computed using the following formula [9]:

Lengthincrement=FinalTotalLength(cm)−PrimaryTotalLength(cm)

$$\text{Survival}\;\text{rate}\;\left(\%\right)=\frac{\text{Number}\;\text{of}\;\text{harvested}\;\text{Fish}}{\text{Number}\;\text{of}\;\text{Fish}\;\text{Stocked}}*100$$

$$\text{ADG}\;\left(\text{Average}\;\text{Daily}\;\text{Gain}\right)=\frac{\text{Number}\;\text{of}\;\text{harvested}\;\text{Fish}}{\text{Duration}\;\text{of}\;\text{Experiment}\;\left(\text{days}\right)}*100$$

$$\text{SGR}\left(\%\right)=\frac{\text{ln}\left(\text{final}\;\text{weight}\right)-\ln\left(\text{initial}\;\text{weight}\right)}{\text{Number}\;\text{of}\;\text{Fish}\;\text{Stocked}}*100$$

#### 4.4.3 Proximate analysis

Proximate analysis of fish was carried out using the dried weight. Thus, fish were dried and finely crushed by mortar and pestle prior to the analyses of crude protein (Kjeldahl Auto System) (ISO 5983–1987), crude lipid (Soxtec HT6) (ISO 6492–1999), moisture (105 °C for 20 h) (ISO 6496–1999), and ash (incineration in a muffle furnace at 540 °C for 16 h) (ISO 5984–2002).

#### 4.4.4 Fatty acid determination

Fatty acids were analyzed by the “Two steps transesterification (2TE)” method after a little modification from Griffiths et al*.* [10]. Lipid was extracted through Digital Soxhlet Apparatus (FOOD ALYTRD40). Methanolic NaOH (1.5 ml) was poured into the lipid extract and mixed in Sonicator (80 °c for 5 mins). After cooling, BF_3_ (2 ml) methanol was added to the mixture and again sonicated (80 °c for 30 min). After cooling, isooctane (1 ml) and saturated NaCl (5 ml) was added and mixed properly. The upper FAMEs (fatty-acid methyl-esters) organic layer was transferred to a new tube and 1 ml sample was taken into injection vial for the analysis by Gas Chromatography Mass Spectrophotometry (GC-2020plus, SHIMADZU, Japan). FAMEs were separated with a capillary column (length 30 m, internal diameter 0.25 mm, film thickness 0.15 µm, phase ratio 250). Helium gas was used (flow rate of 1.42 ml/min) as a carrier gas. The column temperature was set as follows: 180 °C to 280 °C at 5 °C per minute and then held at 280 °C. FAMEs were identified by comparing retention time with standard (FAME mix C8-C24; Sigma- Aldrich; Germany).

#### 4.4.5 Amino acid determination

Slight modification of the Moore and Stein technique [11] was used to assess amino acids. A gram of dried microalgae was hydrolyzed for a day at 110 °C in 25 mL of prepared acidic hydrolysis solution (6 M HCl + 0.1 % phenol). After cooling, a little quantity of SDB/Na (Sample Dilution Buffer) was used to stabilize the sample. The pH of the hydrolysates was adjusted (2.1 to 2.3), filtered and diluted with SDB/Na before placing in the small injection vials. The analysis was performed through SYKAM S 433 amino acid analyzer with UV detector. Nitrogen gas was employed as the carrier gas (0.5 mL/min flow rate, 3 % reproducibility, at 60 °C). Sigma-Aldrich, Germany's AA-S-18 standard wease is used to assess the concentration of final amino acids.

#### 4.4.6 Statistical analysis

The mean ± standard error of all the data were calculated in MS Excel and reported throughout the text. The IBM SPSS (v. 26.0) software was used to perform all statistical analyses related to the survival rate, growth characteristics, proximate and biochemical composition. Descriptive statistics were analyzed for each treatment, followed by a test for homogeneity of variance. A one-way analysis of variance (ANOVA) was performed for data analysis. Significant comparison of treatment groups was performed through Tukey HSD multiple comparison tests at 95 % confidence interval level. Post-hoc test was also performed to discern between treatment group.

## Limitations

Not applicable.

## Ethical Statement

These data were acquired complying ARRIVE guidelines carried out in accordance with the U.K. Animals (Scientific Procedures) Act, 1986 and associated guidelines, EU Directive 2010/63/ EU for animal experiments, or the National Institutes of Health guide for the care and use of Laboratory animals (NIH Publications No. 8023, revised 1978).

## CRediT authorship contribution statement

**Sumit Kanti Dey:** Methodology, Data curation, Writing – original draft. **Jannatul Nayeem:** Data curation, Formal analysis. **Proma Dey:** Data curation. **Abed Hasan Tuser:** Data curation. **Inkiad Ahmed Himel:** Data curation. **Razia Sultana:** Formal analysis. **Mohammed Nurul Absar Khan:** Writing – review & editing. **Helena Khatoon:** Conceptualization, Funding acquisition, Supervision, Resources, Validation, Writing – review & editing.

## Data Availability

Data on growth performance and nutritional composition of common carp (Cyprinus carpio) fry fed with Chlamydomonas sp. (Original data) (Mendeley Data).
